# The reduction of cocoa cake bitterness using natron and its effects on chocolate nutritive value

**DOI:** 10.1002/fsn3.1906

**Published:** 2020-11-24

**Authors:** Roger Ponka, Millie Dauriane Bavoua, Jean Bosco Etoa, Elie Fokou

In Ponka et al. (2020), the important information below should have been added to the article:

The research on which this article finds its origin is a local innovation developed by a farming couple: Valery Ekani Nkoa and his wife Elise in Fegmibang Village in Okola District of Cameroon. Their innovation is documented on the Prolinnova website (www.prolinnova.net) under the title “Utilisation du kanwa pour réduire l'amertume du chocolat” (Utilisation of kanwa to reduce bitterness in chocolate). As part of the project “Promoting local innovation for Food and Nutrition Security” (Proli‐FaNS) funded by the German Government through Misereor/Catholic Central Agency for Development Aid under the umbrella of the Prolinnova network, the farming couple engaged in participatory research with a Masters student, supported by her academic supervisors. They jointly explored questions shared by the local innovators and the researchers about the effectiveness of natron in reducing bitterness in cacao and possible effects on the nutritive value of the chocolate made from it. The process of joint research is documented in: http://www.prolinnova.net/sites/default/files/documents/thematic_pages/food_nutri_sec/proli-fans_farmer-scientist_interaction_280120.pdf

The documentation should ensure that local innovators have the intellectual property rights (IPRs) for their innovations through “copyleft” and that the innovations thus enter into the public domain. The copyleft proviso of Prolinnova is as follows: “Anyone may use the innovation described here and modify or develop it further, provided that the modified or further developed innovation or any follow‐up innovation, of which the innovation described here is an element, is likewise freely available and includes this proviso.”
